# Hybrid Pyramid Convolutional Network for Multiscale Face Detection

**DOI:** 10.1155/2021/9963322

**Published:** 2021-05-05

**Authors:** Shaoqi Hou, Dongdong Fang, Yixi Pan, Ye Li, Guangqiang Yin

**Affiliations:** ^1^School of Information and Communication Engineering, University of Electronic Science and Technology of China, Chengdu, China; ^2^Glasgow College, University of Electronic Science and Technology of China, Chengdu, China; ^3^School of Information and Software Engineering, University of Electronic Science and Technology of China, Chengdu, China

## Abstract

Face detection remains a challenging problem due to the high variability of scale and occlusion despite the strong representational power of deep convolutional neural networks and their implicit robustness. To handle hard face detection under extreme circumstances especially tiny faces detection, in this paper, we proposed a multiscale Hybrid Pyramid Convolutional Network (HPCNet), which is a one-stage fully convolutional network. Our HPCNet consists of three newly presented modules: firstly, we designed the Hybrid Dilated Convolution (HDC) module to replace the fully connected layers in VGG16, which enlarges receptive field and reduces its loss of local information; secondly, we constructed the Hybrid Feature Pyramid (HFP) to combine semantic information from higher layers together with details from lower layers; and thirdly, to deal with the problem of occlusion and blurring effectively, we introduced Context Information Extractor (CIE) in HPCNet. In addition, we presented an improved Online Hard Example Mining (OHEM) strategy, which can enhance the average precision of face detection by balancing the number of positive and negative samples. Our method has achieved an accuracy of 0.933, 0.924, and 0.848 on the Easy, Medium, and Hard subset of WIDER FACE, respectively, which surpasses most of the advanced algorithms.

## 1. Introduction

The face is a key biometric characteristic of humans, thus making face detection the most widely used technology in the field of object detection, recognition, and tracking. The objective of face detection is to detect the existence of a face from a given image and return its size and location, and in practice, many face recognition and pedestrian matching systems have a higher demand for the speed and accuracy of detection.

Because images are taken under a variety of conditions, there is high variability in the scale, occlusion, lighting condition, and viewing angle between faces. To address these problems, the development of face detection techniques underwent three stages: template matching, AdaBoost, and deep learning.

In the early period, most of the face detection algorithms used template matching technology, that is, using a face template image and comparing it with all regions in a given image to judge whether this region contains faces. One representative method was proposed by Rowley et al. [1, 2], who built a multilayer perceptron model using 20 × 20 face and nonface images. Their methods handled the detection of images taken from not only the front [1] but also various angles [2]. Though this model performed well in precision, its detection speed was too slow due to a relatively complex design of classifier and dense sliding-window sampling. After that, machine learning algorithms were used in matching, including neural networks and quorum mode [3], support vector machine based on polemical kernel [4], Bayes classifier [5], and statistics model based on Hidden Markov Models (HMM) [6]. Despite a quite slow speed of detection, these algorithms did not overcome the disadvantages of naïve features.

In 2001, P. Viola and M. Jones published “Rapid Object Detection Using a Boosted Cascade of Simple Features” on CVPR, which represented the coming of AdaBoost period [7]. This Viola–Jones method and Discriminatively Trained Part-Based Models (DPM) [8] were the most commonly known ones in that period. The principle of these algorithms is to build multiplied simple weak classifiers with the help of Haar [9], ACF [10], HOG [11], and other manual features, and then use them to construct a strong classifier possessing a high precision. However, because manual features were a few in number, poor in self-adaption, and stability, these algorithms generally failed to deal with complex conditions like different occlusion, lighting condition, or viewing angles and were usually slow in detection speed.

After that, benefiting from the fast development of deep learning, the above problems were effectively handled. Convolutional neural networks (ConvNet) [12] have a strong expression ability in learning nonlinear features. After its success in image classification, ConvNet was soon applied to face detection and showed a significantly higher precision compared with the previous AdaBoost framework [13]. Cascade CNN [14] can be recognized as a representative of the combination between the traditional method and deep learning. Similar to the algorithms in the Adaboost period, it also adopts a cascade structure, only with ConvNets as its cascade classifiers. Starting from Cascade CNN, a series of deep learning-based object detection algorithms were proposed. The most representative ones include one-stage algorithms with a fast speed, such as YOLO series [15–17], SSD series [18–20], and two-stage algorithms with high precision, such as Fast R-CNN series [21, 22], MTCNN [23], and R-FCN series [24]. These general detection models were also applied to face detection and performs well.

However, though most deep learning algorithms achieve success under different lighting conditions and viewing angles, their performances are still disappointing when confronted with complex circumstances like multiscale and occlusion. By comparing these methods, we found that one common drawback of them is to use a single or simple composite feature map rather than combine semantic information from higher layers together with details from lower layers effectively. For example, most two-stage ConvNets use several single feature maps and ignore information from higher or lower layers, while ScaleFace [25] combines features from only the lower layers. We assumed that this was the main reason for these methods to fail under extreme conditions.

In this paper, we proposed a multiscale face detection algorithm based on HFP structure and design a new face detection framework HPCNet. The main contributions of this paper are concluded as follows:

  Targeting large-scale detection, we designed a HDC module, which can enlarge receptive field (RF) rapidly to acquire feature maps with a higher resolution. This mechanism is introduced from object segmentation to face detection for the first time.
  Targeting small-scale detection, we presented a HFP structure as the core of our model, which combines semantic information from higher layers together with details from lower layers. Compared with Feature Pyramid Network (FPN) [26], HFP processes features more carefully, with more convolution operations before feature fusion.
  Targeting face occlusion and blurring, we introduced a CIE module here, which reduces the amount of computation and avoid feature confusion.

In addition, in the training stage, we presented an improved OHEM strategy in face of the imbalance between the number of positive and negative samples and introduced multiscale training to enhance the robustness of the model further. After running on the authoritative WIDER FACE [27], we found that our model showed a high precision of 0.933, 0.924, and 0.848 on three subsets Easy, Medium, and Hard, respectively. When running on GTX 1080Ti, the inference speed can achieve 44 Frames Per Second (FPS) with a higher resolution. After a series of comparative experiments, we proved our method to be reasonable.

The rest of the paper is organized as follows: Section 2 introduces some related works.  Section 3 illustrates the proposed methods from point to total.  Section 4 provides the experiments and Section 5 concludes the paper.

## 2. Related Works

### 2.1. Dilated Convolution

SSD [18], SFD [28], DSFD [29], and other algorithms add several convolution layers at the end of VGGNet [30] to address with large-scale objective or face. These added convolution layers help to process the information further, reduce the size of the feature map, and enlarge RF. Dilated Convolution has similar effects, only with the size of the feature map unchanged.

Specifically, dilated convolution is to make convolution kernel dilate. Assuming that the size of the kernel is *f* × *f* and the dilation factor is *d*, then the size of the kernel after dilation *f*_*d*_ is

(1) $$\begin{array}{c} f_{d}=1+\left(f-1\right)d. \end{array}$$

The number of inserted pixels *p*_*d*_ is

(2) $$\begin{array}{c} p_{d}=\left\lfloor \frac{f_{d}}{2}\right\rfloor . \end{array}$$

**Figure 1:**
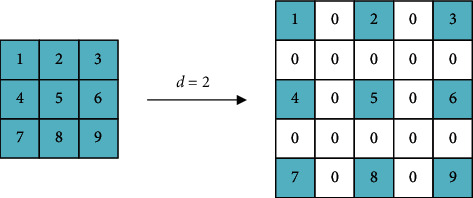
The dilation process of the convolution kernel.

The dilation process of the kernel is shown in Figure 1, where the blank space left after dilation is filled by 0. Dilation convolution can enlarge kernel and RF rapidly without changing the size of the feature map, thus generating a feature map of a higher resolution. Dilated convolution is also commonly used in extracting structured and context information.

### 2.2. Feature Pyramid

**Figure 2:**
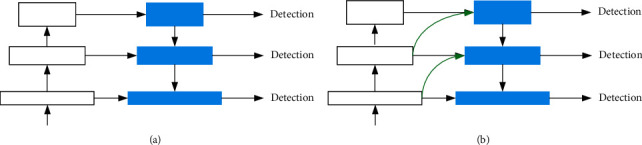
The simplified structure of the feature pyramid before and after improvement. (a) Feature Pyramid Network (FPN). (b) Hybrid Feature Pyramid (HFP).

To use feature maps of different scales for object detection is an effective method to handle the scale problem. There are mainly two ways to realize it: one is the featured image pyramid [31] and another one is using multiscale feature maps at the end of the network (as shown in Figure 2(a)). The former one has a large amount of computation due to repetitive calculation and has difficulty training network in an end-to-end way, while the latter one avoids this successfully. Nevertheless, neither of these two methods takes advantages of feature maps from higher layers. As feature maps in lower layers contain no semantic information, its absence brings challenges to detection.

Our objective is to take full advantage of the pyramidal feature hierarchy embedded in ConvNets, which contains information from lower to higher layers and construct a feature pyramid combining information from lower to higher layers together.

**Figure 3:**
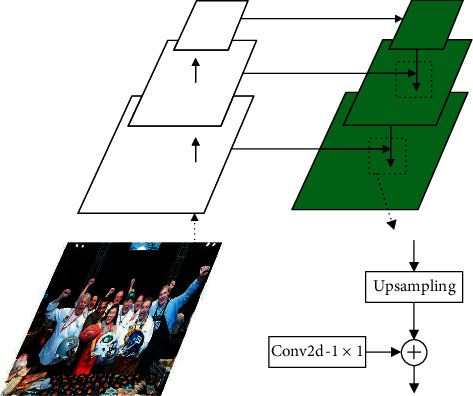
The structure of FPN.

FPN [26] offers a rather simple way to use feature maps. Its principle is to build a top-down architecture by introducing higher-level information to the current layer: first, the feature map from higher pyramid levels is upsampled by a factor of 2 (using nearest neighbor upsampling for simplicity); then, it undergoes a 1 × 1 convolutional layer to reduce channel dimensions; finally, the upsampled map is merged with the current map (which undergoes a 1 × 1 convolutional layer) by elementwise addition. The detailed process of merging is shown in Figure 3.

### 2.3. Context Information

When humans search for faces, they take not only faces but also hats, clothes, surroundings, and other information. Context information is exactly a simulation of this behavior. When it is difficult to judge whether the candidate proposal contains faces, we can use the information around the proposal as a supplement, which is an effective way to handle occlusion and blurring.

Based on the experience, CMS-RCNN [32] combined face and body information together for face detection. The spatial relationship between face and body is described as follows:

(3) $$\begin{array}{c} t_{x}=\frac{x_{b}-x_{f}}{w_{f}},\\ t_{y}=\frac{y_{b}-y_{f}}{h_{f}},\\ t_{w}=\log\frac{w_{b}}{w_{f}},\\ t_{h}=\log\frac{h_{b}}{h_{f}}, \end{array}$$

where *f* and *b* represent face and body, respectively; *t* is a fixed value; *x*, *y*, *w*, and *h* represent the center coordinate, width, and height of candidate proposal. CMS-RCNN substitutes the coordinate of extracted face candidate into equation (3) to acquire body candidate and then maps face and body candidate onto feature maps. After undergoing pooling layer, convolutional layer, and two fully connected layers, they are joint together for bias regression and classification of coordinates. The way that CMS-RCNN acquires context information can be easily combined with the two-stage objective detection algorithm, which is credited to the RoI pooling layer. Though the CIE in CMS-RCNN has positive effects on the detection result, the assumption it contains is too strong to be accurate and it is difficult to combine with one-stage detection algorithms.

### 2.4. OHEM

OHEM [33] is a completely online hard sample mining algorithm, which samples according to the nonuniform and nonstationary distribution depending on sample classification loss, and makes simple changes to the stochastic gradient descent. For each detection task, OHEM chooses *N* samples with a higher loss from thousands of proposals or anchors in one or two images. Though only using a part of proposals or anchors, its backward propagation is still effective and robust. The reason why OHEM does not use all the samples is that simple samples contribute little to the loss. In addition, when there are too many negative samples, the dataset is filled with simple samples, which altogether have a huge effect on loss, with no help to classification. The same with SVM, it is the hard samples that contribute truly to classification. Compared with hard sample mining, OHEM does not need to construct a dataset or train model; while compared with stochastic gradient descent, OHEM takes advantage of hard samples that make a contribution to classification loss and thus avoid useless computation.

## 3. Method

In this section, we will introduce each proposed module and give a comprehensive description of the overall framework of HPCNet.

### 3.1. Components in HPCNet

#### 3.1.1. HDC Module

Instead of adding several convolution layers at the end of a basic convolution network like SSD [18], SFD [28], and DSFD [29], we introduce the concept of dilated convolution to handle large-scale faces, which is a new trail.

There exist some drawbacks in the common dilated convolution. Assume that there is a pixel *v* in the *l* th layer, and the *f*_*d*_ × *f*_*d*_ area that contributes to *v* is in the *l* − 1 th layer around the location of *v*. Because the dilated kernel introduced several 0, the actual contribution area is still *f* × *f*. As the dilation factor increases, the contribution area in *l* − 1 th layer enlarges rapidly, while the real contribution area stays the same. Therefore, the local feature information gradually gets lost due to 0 values, and the correlation of information contributing to *v* decreased consistently. When several dilated convolution layers are connected in series, this effect will be exacerbated continuously.

**Figure 4:**
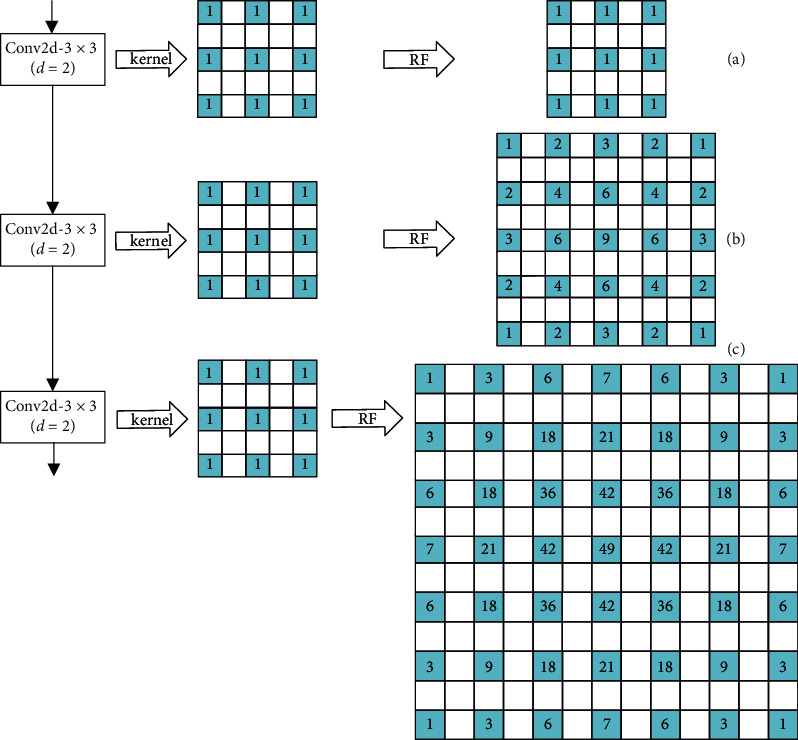
An illustration of the area that truly contributes to *v* in three series-connected layers with the same factor of 2.

Assume that there are three dilated convolution layers forming a structure *s*, where the kernel is 3 × 3, dilation factor is 2, and the sliding stride is 1. With structure *s* replacing the *l* th layer, the RF area that truly contributes to *v* in the *l* − 1 th layer is shown in Figures 4(a)–4(c). The number in the blue grid represents its contribution value and the white grid has no contribution. The value in Figure 4 is calculated under the assumption that the values of the kernel and the *l* − 1 th feature map are all 1.

**Figure 5:**
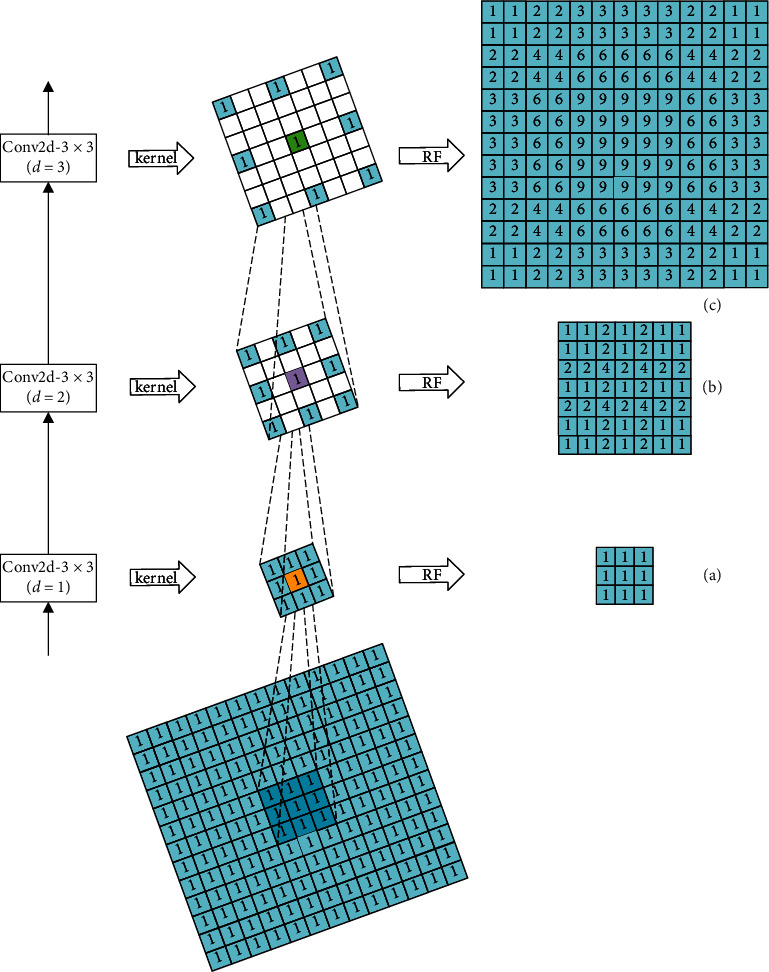
An illustration of the area that truly contributes to *v* when using HDC.

To make use of the advantages of dilated convolution, as well as avoiding local information loss and correlation reduction, we designed the HDC module. HDC only contains three dilated convolution layers, of which the kernel size is 3 × 3, dilation factor is 1, 2, and 3, respectively, and sliding stride is 1. With HDC replacing the *l*th layer, the RF area that truly contributes to *v* in the *l* − 1 th layer is shown in Figures 5(a)–5(c). It is clear from Figure 5 that, in every stage, all the grids in RF area contribute to *v*, and the weights of which increase as getting closer to the location of *v*. This structure is obviously reasonable.

#### 3.1.2. HFP Module

Though FPN [26] introduced semantic information from the higher layer into the current feature map, there still exist three problems:

  FPN generates composite feature map by elementwise addition, which lacks self-adaption and can easily cause feature confusion
  FPN ignores information from lower layers when constructing a feature map, which results in a lack of details and location information, thus bringing a challenge to locating and detecting small-scale objects
  The composite feature map obtained is used both as high-level semantic information and for detection, which is not a reasonable way as it undertakes too many tasks

Our HFP (as shown in Figure 2(b)) is an improvement of FPN targeting at the above three problems. A summary of its process is as follows: first, it upsamples feature maps from higher layers (using bilinear interpolation) and reduces their channel dimensions to generate composite feature maps by merging with current ones; then the composite feature map is further processed to obtain truly useful semantic information by reducing channel dimensions; after that, similarly, downsampling and channel dimensions reduction are applied to feature maps from lower layers to acquire hybrid feature maps by stitching with composite ones; finally, the hybrid feature maps are used for detection after channel changes and information fusion.

**Figure 6:**
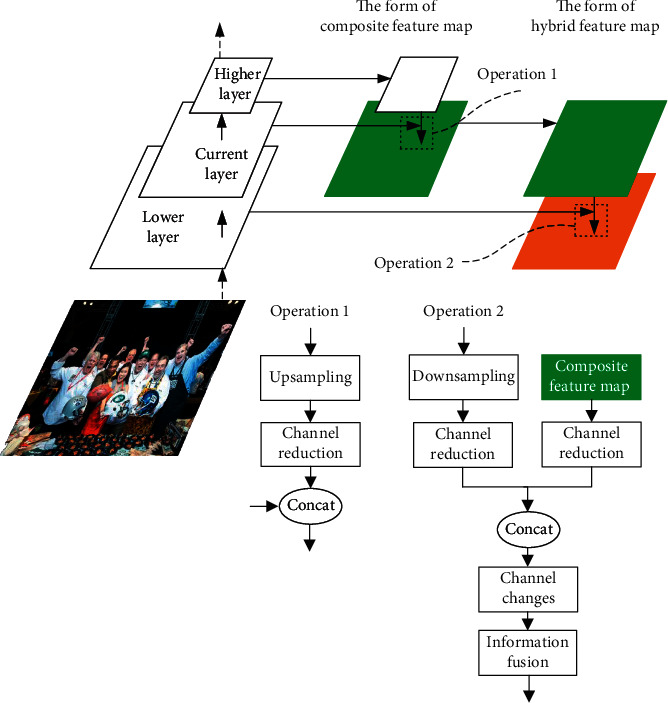
An illustration of the detailed structure of HFP.

To be more specific, the details in HFP (as shown in Figure 6) are as follows: for high-level feature maps, we use 1 × 1 convolution layer for channel dimension reduction, as the 1 × 1 kernel will not change RF and is more suitable for semantic learning. For composite feature maps, we use 3 × 3 convolution layer when used as high-level semantic information, as the 3 × 3 kernel can avoid feature confusion resulting from upsampling and downsampling. For low-level feature maps, we use 3 × 3 convolution layer with a stride of 2 for downsampling in order to save detailed information; then the feature maps undergo another 3 × 3 convolution layer for channel dimension reduction to extract truly needed details. For hybrid feature maps, we use 3×3 convolution layer for channel changes and information fusion as well.

Our HFP is different from FPN in several aspects:

  In HFP, composite feature map is generated by channel joint, while FPN uses elementwise addition instead.
  The processing procedure of feature maps for detection is different. In FPN, composite feature maps are used in detection directly, while our HFP processes composite ones further and combines them with low-level ones before detection.
  HFP handles feature maps in a more careful way and adopts a series of dimension operations to acquire effective information.

#### 3.1.3. CIE Module

Despite enlarging the window around the candidate proposals, a bigger kernel is a better choice for a one-stage object detection algorithm to obtain information around faces.

**Figure 7:**
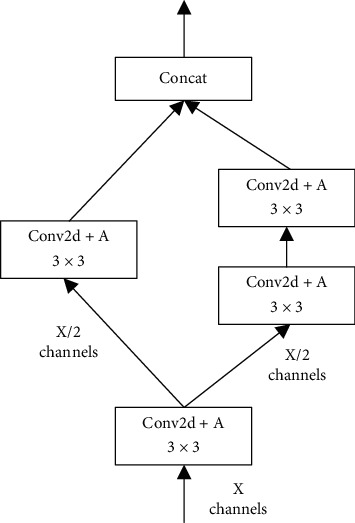
An illustration of the detailed structure of CIE.

SSH [34] adopts this strategy by applying simply two bigger kernels to extract context information. However, a bigger kernel usually leads to a bigger amount of computation, which can be replaced by several smaller ones connected in series. Inspired by this idea and SHH, we proposed our CIE which only contains convolution layers with 3 × 3 kernels. To reduce the amount of computation further and prevent the correlation of context from decreasing, we adopt a method to share some convolution layers. The detailed structure is shown in Figure 7.

#### 3.1.4. Improvements of OHEM

Though OHEM [33] is robust and highly efficient, it only considers hard samples without taking the ratio of positive to negative samples into consideration. As a large number of samples in the dataset are negative, the ones chosen by OHEM may also suffer from an imbalance of two samples, which is obviously detrimental to classification. Therefore, we proposed an improved OHEM, which chooses samples in a more balanced way: assuming that the loss function needs *N* samples, first, we sort positive and negative samples in descent order by loss, respectively; then, we choose the first *S* positive ones and *N* − *S* negative ones. The default value of *S* is set as *N*/4. In the ideal case, there are *N* samples chosen with a ratio of 1 : 3 [22]. Even if the total number is less than *N* and the ratio is not 1 : 3 exactly due to a lack of positive samples, these usually will not do harm to the performance of the algorithm. In contrast, they can enhance the robustness of the algorithm.

### 3.2. The Overall Layout of HPCNet

HPCNet is a one-stage multiscale face detection algorithm. To handle large-scale and small-scale faces, HPCNet introduced HDC module and HFP structure; to address with occlusion and blurring, HPCNet contained CIE.

**Figure 8:**
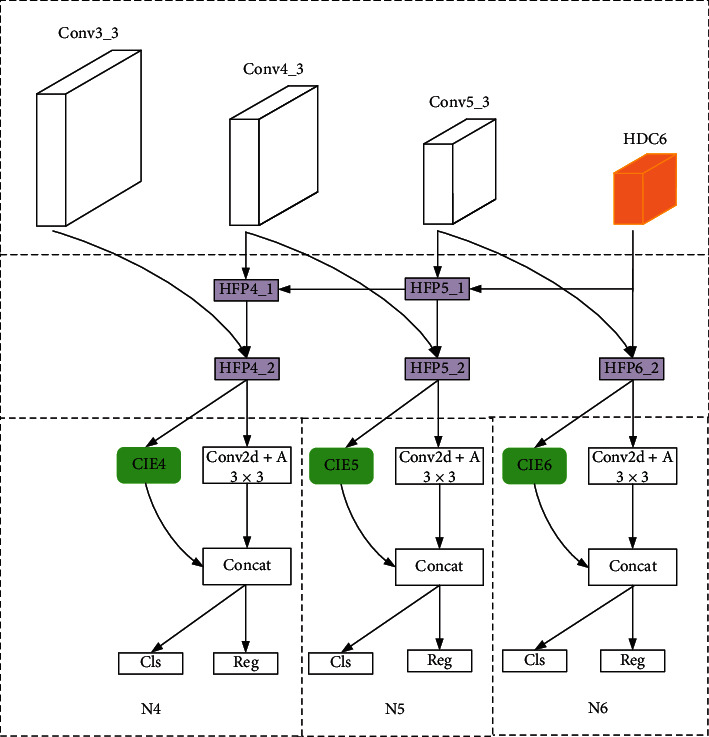
The overall layout of HPCNet.

HPCNet contains the convolution layers in VGG16 [30] as its basic network (as shown in Table 1). The overall structure is shown in Figure 8, where N4, N5, and N6 are three subnetworks for detecting different scales of face, namely, small, medium, and large. One thing that should be noticed here is that all the convolution layers we used are 3 × 3, as they cut down on parameters and computation in addition to satisfying needs for processing.

**Figure 9:**
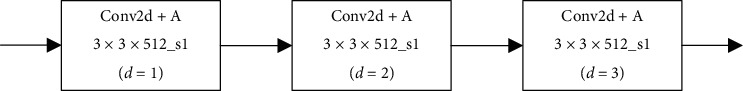
The structure of HDC6.

In Figure 8, HDC6 refers to the proposed HDC module, which keeps in line with the architecture of VGG16 (as shown in Figure 9).

**Figure 10:**
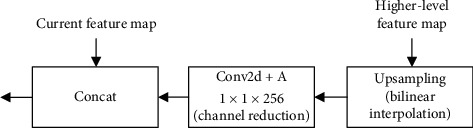
The structure of HFPx_1.

**Figure 11:**
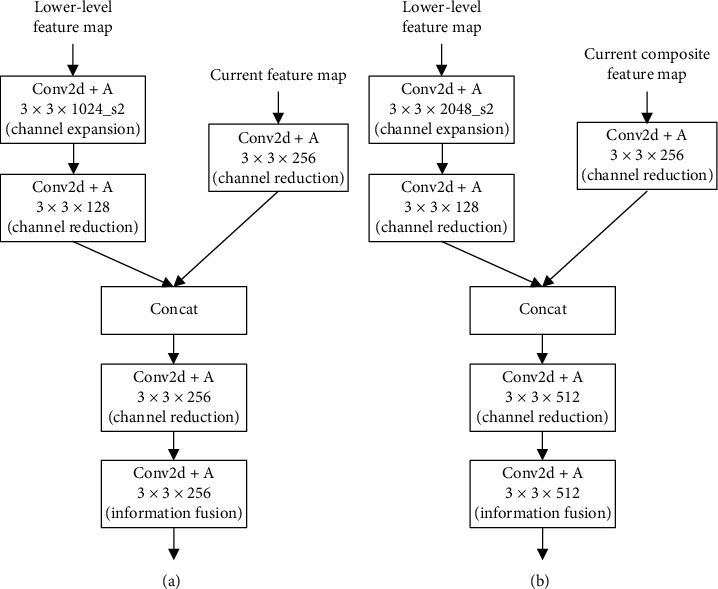
The structure of HFPx_2. (a) HFP4 2. (b) HFP5_2 and HFP6_2.

HFP module consists of HFPx_1 (including HFP4_1 and HFP5_1) and HFPx_2 (including HFP4_2, HFP5_2, and HFP6_2). HFPx_1 (as shown in Figure 10) is to generate composite feature maps, which are passed forward as high-level semantic information; HFPx_2 is to generate hybrid feature maps, which combines information from both high and low layers. HFP4_2 (as shown in Figure 11) uses 3 × 3 convolution layer to reduce channel dimension of hybrid feature maps to 256, while HFP5_2 and HFP6_2 increase channel dimension to 512. The reason for this difference in HFP4_2 is to reduce memory occupation and keep in line with the following modules.

CIE4, CIE5, and CIE6 are three individual CIE modules, the structures of which are shown in Figure 7. Each subnetwork contains one CIE module as one branch and one 3 × 3 convolution layer as another. The feature maps for classification are generated by two branches together, the channel dimension of which is half that in HFPx_2, respectively (as shown in Figure 8). In N5 and N6, the channel dimension through CIE5 and CIE6 is 256, while in N4, the channel dimension through CIE4 is 128. The reason for fewer channels in CIE4 is to reduce memory occupation and accelerate network convergence.

## 4. Experiment and Analysis

In this section, we first introduced some training strategies and parameter settings of HPCNet. Then, we conducted a series of ablation experiments on the WIDER FACE [27] dataset and compared HPCNet with other advanced algorithms to prove the effectiveness of our method.

### 4.1. Training Details

#### 4.1.1. Dataset

All the experiments in this paper are based on WIDER FACE [27], which is the largest and most authoritative face image dataset in the world. In WIDER FACE, there are 32203 images containing 393703 labeled faces, which is of high variability in terms of scales, occlusion, posture, and other aspects. In this paper, we randomly choose 40%, 10%, and 50% of the dataset as training, validation, and test set, respectively. In each set, the data is divided into three subsets (viz., Easy, Medium, and Hard) according to the difficulty level of detection.

Before entering into HPCNet, all the images are scaled to less than *S* × *L*. To be more specific, we first scale the height of images to *S* pixels. After that, if its width is longer than *L* pixels, the width of this image is scaled to *L* pixels. During the scaling, the aspect ratio of all images remains unchanged.

#### 4.1.2. Hard Example Mining

The feature maps generated by three subnets N4, N5, and N6 correspond to 8 × 8, 16 × 16, and 32 × 32 area in the original image. The prior anchors used in *N*4, *N*5, and *N*6 are 16 × 32, 64 × 128, and 256 × 512, respectively. All of them have an aspect ratio of 1. During the training, we set the candidate proposals with Intersection over Union (IoU) higher than 0.5 as positive samples, while the ones with IoU lower than 0.3 as negative samples. After that, we process the dataset with an improved OHEM strategy.

#### 4.1.3. Loss Function

To handle the problems of classification and regression at the same time, HPCNet adopts multitask loss function, which can be represented as

(4) $$\begin{array}{c} L_{\text{total}}=\underset{i}{\sum }\left(\frac{1}{N_{i}}\underset{j\in B_{i}}{\sum }L_{\text{conf}}\left(p_{j},g_{j}\right)+\frac{\lambda }{N_{i}}\underset{j\in B_{i}}{\sum }I\left(g_{j}=1\right)L_{\text{loc}}\left(b_{j},t_{j}\right)\right), \end{array}$$

where *L*_total_ represents the total loss, *L*_conf_ represents the classification loss, and *L*_loc_ represents the regression loss. For *L*_conf_, we use *Softmax* function targeting at binary classification, in subnet *N*_*i*_, *N*_*i*_ represents the number of samples, *B*_*i*_ represents the whole dataset, and *p*_*j*_ and *g*_*j*_ represent the class score and label of *j* th sample. For *L*_loc_, we use Smooth_*L*1_ function with *I* representing the characteristic function: if the *j*th sample in subnet *N*_*i*_ is positive (i.e., *g*_*i*_=1), then *I*=1; otherwise *I*=0. Here, *b*_*j*_ and *t*_*j*_ represent the coordinate prediction and preset value of the *j*th sample in subnet *N*_*i*_; *λ* controls the ratio of *L*_conf_ to *L*_loc_ (set as 1). If there is no positive sample in subnet *N*_*x*_, *L*_loc_ is set as 0.

#### 4.1.4. Hyperparameter Setting

The weights in HPCNet are initialized by Gaussian function with an average of 0 and variance of 0.01. The bias is initialized as 0 and the regularization parameter is set as 0.0005. The training process adopts a batch SGD algorithm with a momentum of 0.9, itersize as 2, batchsize as 1, and initial learning rate as 0.004 (adopting StepLR policy with Gamma of 0.1 and stride as 18,000). Our HPCNet uses four GTX 1080Ti GPU to train for 21,000 times in total.

### 4.2. Ablation Experiment and Results

#### 4.2.1. Analysis of Improved OHEM

We trained HPCNet with OHEM and improved OHEM, respectively, the result of which on WIDER FACE is shown in Table 2.

The average precision (AP) of improved OHEM on Hard subset is 2.4% higher than that of OHEM, though it is 0.4% and 0.2% lower on Easy and Medium subsets. As Hard subset contains the most difficult cases which is closer to a real application, it is proved that improved OHEM is better. All the following experiments adopt improved OHEM.

#### 4.2.2. Analysis of HDC Module

To test the effects of the HDC module, we get rid of HDC6 in HPCNet and named the network HPCNet-HDC6. The result is shown in Table 3. HPCNet-HDC6 is 1.6%, 1.2%, and 1.2% lower than HPCNet in terms of AP on each subset. Compared with the other two subsets, AP on Easy subset shows a bigger decrease, which proves that HDC6 is effective especially in detecting large-scale faces.

#### 4.2.3. Analysis of HFP Module

**Figure 12:**
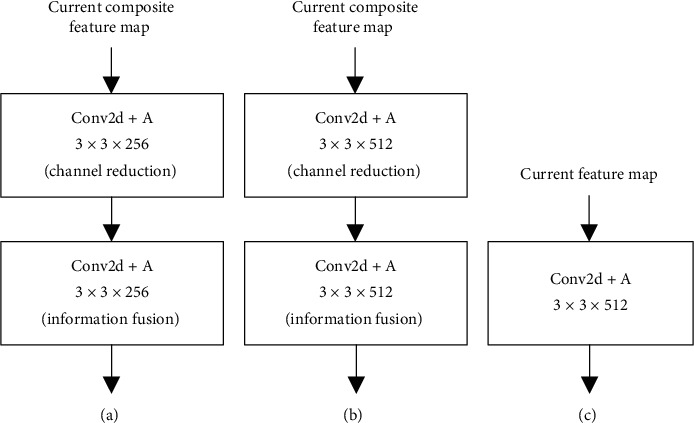
The structure of HFPx_2. (a) HFP4_2. (b) HFP5_2 and HFP6_2. (c) HFP5_2 and HFP6_2.

To clearly illustrate the importance of low-level detailed information in HFP, we changed the architecture of HFPx_2 by deleting the feature maps from lower layers. The changed HFPx is shown in Figure 12 and the network is named as HPCNet-Lx. From Table 3, we can see that HPCNet-Lx shows an obvious lower AP on all subsets, with an emphasis on the Hard subset (from 81.9% to 79.5%, reduced by 2.4%). This result has proved that low-level feature maps are essential to small-scale face detection.

#### 4.2.4. Analysis of CIE Module

In this experiment, we removed the CIEx in HPCNet and set the number of channels in the main branch as the total number (256 in N4, 512 in N5, and N6). The changed network is named as HPCNet-CIEx. It is clear from Table 3 that HPCNet-CIEx is 1.6%, 1.2%, and 0.8% lower than HPCNet in terms of AP on each subset, which shows the effect of CIE on large-scale occlusion problems.

#### 4.2.5. Analysis of Multiscale Training

**Figure 13:**
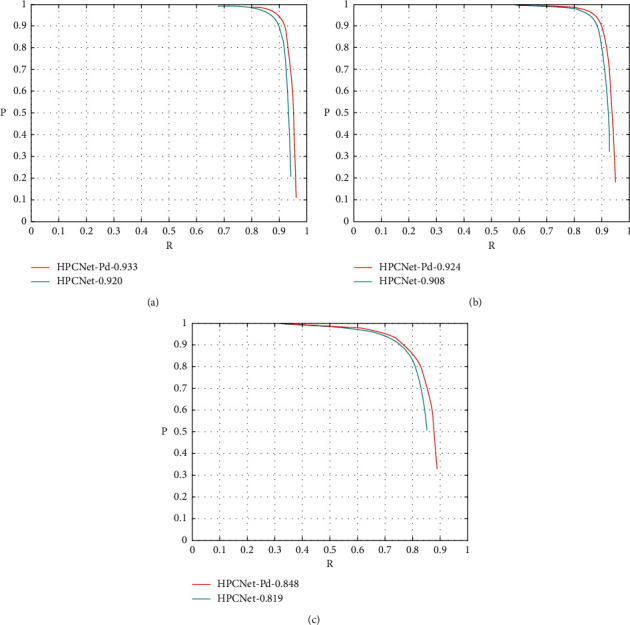
The PR curves of HPCNet and HPCNet_Pd on each subset. (a) Easy, (b) Medium, and (c) Hard.

We adopt multiscale training to HPCNet. To be more specific, it is randomly scaling images to 400 × 1600, 600 × 1600, 800 × 1600, 1000 × 1600, 1200 × 1600, 1400 × 1600, and 1600 × 1600. All of these sizes follow the principle of *S* × *L*. The result is shown in Table 4 and Figure 13. We name the model after multiscale training as HPCNet_Pd.

From Table 4, we can see that the AP of HPCNet_Pd on each subset is 1.3%, 1.6%, and 2.9% higher than HPCNet. The reason for this improvement is that multiscale training is indeed an image boosting strategy, which generates more faces of different scales and therefore enhances adaption and robustness of the model.

#### 4.2.6. Comparison with Other Algorithms

We choose several face detection algorithms to compare with HPCNet, namely, two-stage CNN [22], MTCNN [23], ScaleFace [25], SFD [28], DSFD [29], CMS-RCNN [32], SSH [34], HR [35], and FacenessNet [36]. The reason for choosing them is as follows:

  All of them are based on ConvNet
  They are representative in different genres
  They have a good performance on WIDER FACE
  They take both precision and time into consideration

**Figure 14:**
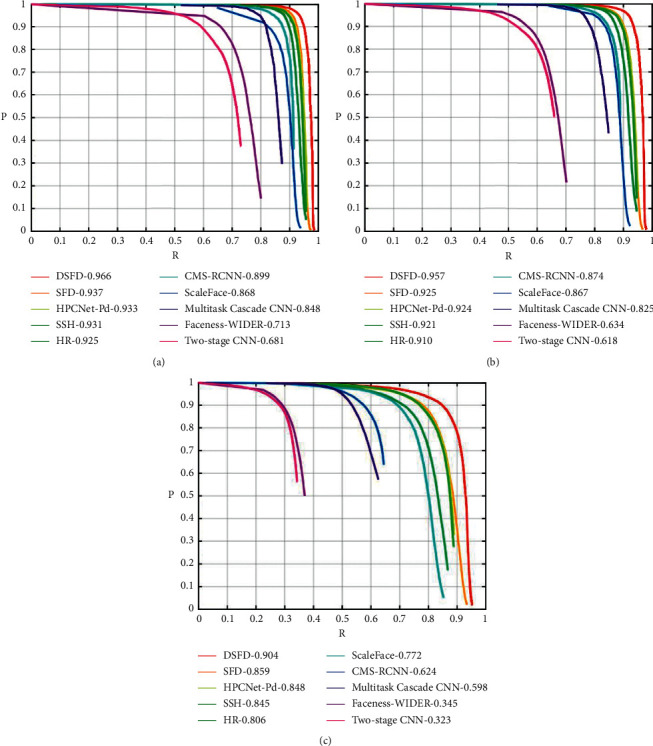
The PR curves of all the algorithms on WIDER FACE subset. (a) Easy, (b) Medium, and (c) Hard.

The comparison result is shown in Figure 14 and Table 5. Despite HPCNet, all the AP and curve data are from the website of WIDER FACE [27]. Figure 14 directly shows differences between algorithms, where *R* represents recall rate and *P* represents precision.

**Figure 15:**
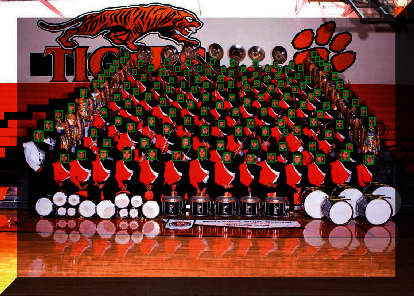
An illustration of detection result using HPCNet.

It is clear from Table 5 that HPCNet has a higher AP on three subsets than classical algorithms including two-stage, FacenessNet, MTCNN, ScaleFace, CNNCMS-RCNN, HR, and SSH. For the most advanced algorithms like SFD and DSFD, though HPCNet shows slightly lower AP, its running speed is much faster. The result has shown that HPCNet can have an advanced detection rate as well as running speed, which proves its reasonability and effectiveness.  Figure 15 is an example of small-scale face detection by HPCNet.

## 5. Conclusion

Scaling and occlusion are the most challenging problems for face detection currently. We conducted research targeting these difficulties and proposed a one-stage, fully convolutional face detection framework HPCNet, which contains several designed components. In HPCNet, we introduced the concept of HDC and enlarged RF to handle large-scale faces. Meanwhile, we proposed a new HFP structure combining high-level and low-level features together to enhance performance on small-scale faces. In addition, aimed at occlusion, we designed the CIE with fewer parameters. Particularly, we took advantage of improved OHEM and multiscale training strategy to balance the number of different samples as well as enhance robustness. By a series of ablation experiments, we proved the superiority of our HPCNet. In the future, the idea of this method can be applied to other computer vision tasks, such as person reidentification.

## Figures and Tables

**Table 1:** The basic structure of HPCNet.

Name	Configurations
Conv1_1	Conv2d + *A*, 3 × 3 × 64_s1
Conv1_2	Conv2d + *A*, 3 × 3 × 64_s1
Downsampling	M*A*xpool, 2 × 2_s2
Conv2_1	Conv2d + *A*, 3 × 3 × 128_s1
Conv2_2	Conv2d + *A*, 3 × 3 × 128_s1
Conv2_3	Conv2d + *A*, 3 × 3 × 128_s1
Downsampling	M*A*xpool, 2 × 2_s2
Conv3_1	Conv2d + *A*, 3 × 3 × 512_s1
Conv3_2	Conv2d + *A*, 3 × 3 × 512_s1
Conv3_3	Conv2d + *A*, 3 × 3 × 512_s1
Downsampling	M*A*xpool, 2 × 2_s2
Conv4_1	Conv2d + *A*, 3 × 3 × 512_s1
Conv4_2	Conv2d + *A*, 3 × 3 × 512_s1
Conv4_3	Conv2d + *A*, 3 × 3 × 512_s1
Downsampling	M*A*xpool, 2 × 2_s2
Conv5_1	Conv2d + *A*, 3 × 3 × 512_s1
Conv5_2	Conv2d + *A*, 3 × 3 × 512_s1
Conv5_3	Conv2d + *A*, 3 × 3 × 512_s1
Conv6	Conv2d + *A*, 3 × 3 × 512_s2
HDC6	Conv2d + *A*, 3 × 3 × 512_s1_d1
	Conv2d + *A*¸ 3 × 3 × 512_s1_d2
	Conv2d + *A*, 3 × 3 × 512_s1_d3

**Table 2:** The result of OHEM and improved OHEM.

Name	Easy	Medium	Hard
OHEM	0.924	0.910	0.795
Improved OHEM	0.920	0.908	0.819

**Table 3:** The effect of different components.

Name	Easy	Medium	Hard
**HPCNet**	**0.920**	**0.908**	**0.819**
HPCNet-HDC6	0.904	0.896	0.807
HPCNet-Lx	0.912	0.900	0.795
HPCNet-CEx	0.904	0.896	0.811

**Table 4:** Analysis of multiscale training.

Name	Easy	Medium	Hard
HPCNet	0.920	0.908	0.819
**HPCNet_Pd**	**0.933**	**0.924**	**0.848**

**Table 5:** Comparison between HPCNet and other algorithms.

Name	Easy	Medium	Hard	FPS
Two-stage CNN	0.681	0.618	0.323	<5
FacenessNet	0.713	0.634	0.345	<100
MTCNN	0.848	0.825	0.598	<95
ScaleFace	0.868	0.867	0.772	<5
CMS-RCNN	0.899	0.874	0.624	<15
HR	0.925	0.910	0.806	<5
SSH	0.931	0.921	0.845	<15
SFD	0.937	0.925	0.859	<35
DSFD	0.966	0.957	0.904	<20
**HPCNet_Pd**	**0.933**	**0.924**	**0.848**	**44**

## Data Availability

The previously reported data were used to support this study and are available at 10.1109/CVPR.2016.596. These prior studies and datasets are cited at relevant places within the text as [27].
